# Investigation of the Carbonation Behavior of Cement Mortar Containing Interior Stone Sludge and Recycled Mask Fibers

**DOI:** 10.3390/ma18225218

**Published:** 2025-11-18

**Authors:** Junhyeok Choi, Seongjin Cho, Dongkyu Lee, Gwang Mok Kim, Beomjoo Yang, Daeik Jang

**Affiliations:** 1 School of Civil Engineering, Chungbuk National University, 1 Chungdae-ro, Seowon-gu, Cheongju 28644, Republic of Korea; chlwnsgur4512@cbnu.ac.kr (J.C.); sjini0515@cbnu.ac.kr (S.C.); jayjaycorp@hanmail.net (D.L.); 2 Mineral Processing & Metallurgy Research Center, Resources Utilization Division, Korea Institute of Geoscience and Mineral Resources, 124 Gwahak-ro, Yuseong-gu, Daejeon 34132, Republic of Korea; k.gm@kigam.re.kr; 3 Department of Civil Engineering, The University of Texas at Arlington, Arlington, TX 76019, USA

**Keywords:** interior stone sludge, recycled mask fiber, carbonation curing, thermal performance, cement mortar

## Abstract

**Highlights:**

**What are the main findings?**

- This study explores the use of interior stone sludge and recycled mask fibers in cement mortars.
- The proposed approach aims to improve sustainability and reduce environmental impact.

**What is the implication of the main finding?**

- Sludge acts as a fine filler that increases matrix compactness.
- Mask fibers lower thermal conductivity and help mitigate cracking.

**Abstract:**

This study examines the carbonation and mechanical behavior of cement mortar incorporating artificial interior stone (AIS) sludge and recycled mask fibers (RMFs). Sludge, derived from AIS waste, replaced 30 wt.% of fine aggregate, while RMF from polypropylene masks was added at 0–1 wt.% of cement. Specimens were cured under normal and carbonation conditions (10% CO_2_, 25 °C, 60% RH) for 7 and 28 days. Carbonation curing improved compressive and flexural strengths by up to 28% and 88%, respectively, and enhanced microstructural densification. Although the incorporation of AIS sludge reduced compressive strength due to its inert and irregular particle characteristics, it effectively refined the pore structure and decreased overall porosity. The inclusion of RMF at moderate contents (0.25–0.5 wt.%) improved crack resistance and lowered thermal conductivity, demonstrating a favorable balance between strength and thermal performance. TGA/DTG results confirmed increased CaCO_3_ formation and greater CO_2_ uptake. After exposure to 500 °C, carbonation-cured mortars retained higher residual strength, indicating superior thermal stability.

## 1. Introduction

Global efforts to mitigate greenhouse gas emissions have intensified in response to climate change and environmental degradation [1,2,3]. Among industrial sectors, cement production is recognized as one of the major contributors to anthropogenic CO_2_ emissions, generating nearly 900 kg of CO_2_ per ton of cement produced. This has prompted growing interest in carbon capture, utilization, and storage (CCUS) technologies that can both reduce and reuse CO_2_ within construction materials [4,5].

In parallel, the construction industry faces mounting challenges associated with the disposal of industrial and plastic waste [6,7,8]. The production of artificial interior stone, a popular finishing material, generates a large amount of sludge composed mainly of quartz and polymeric residues [9]. This sludge is typically landfilled or incinerated, causing secondary pollution [10]. Similarly, the global surge in single-use polypropylene masks since the COVID-19 pandemic has led to a sharp increase in plastic waste, with most discarded masks being incinerated or buried, releasing toxic gases and microplastics.

Previous studies have shown that fine mineral powders such as artificial interior stone (AIS) sludge can effectively act as micro-fillers in cementitious systems, improving particle packing and mechanical performance. Kim et al. [11] demonstrated that replacing 15–30 wt.% of fine aggregate with AIS sludge reduced total porosity and critical pore size, leading to enhanced compressive strength. This improvement was attributed to the sludge’s narrow particle size distribution and its high SiO_2_ content, which promotes a filler-induced densification rather than a pozzolanic reaction [12]. Similar findings were reported by Choi et al. [13], who observed that AIS sludge shortened setting time and refined the pore structure due to its nucleation effect during hydration. The quartz-rich and chemically inert nature of AIS sludge makes it particularly effective in mitigating large pore formation, thereby enhancing durability and dimensional stability under elevated temperatures. However, most existing studies focused on normal curing conditions, and the potential of carbonation curing to further enhance these microstructural benefits has not been extensively examined.

In contrast, research on recycled mask fibers (RMFs) derived from discarded polypropylene masks has mainly emphasized their role as fiber reinforcements in cement-based materials [14]. Win et al. [15] and Miah et al. [16] reported that incorporating 0.10–0.25 vol.% of RMF improved flexural and tensile strength through crack-bridging effects, while excessive fiber content (≥0.5%) caused fiber agglomeration, poor workability, and increased porosity, ultimately reducing compressive strength. These findings are consistent with broader studies on polypropylene fibers in mortar and concrete, which highlight the need for optimal fiber dosage and surface compatibility [17]. Meanwhile, carbonation curing has been proven to significantly enhance strength, CO_2_ uptake, and microstructural densification by forming CaCO_3_ within the matrix [18,19]. Despite these advancements, few studies have investigated the combined use of AIS sludge and RMF under accelerated carbonation.

Therefore, this study aims to comprehensively evaluate the effects of AIS sludge and RMF on the carbonation behavior, mechanical performance, and thermal properties of cement mortar. While previous studies have typically focused on the use of mineral waste powders or polymer fibers independently, this work uniquely investigates the combined utilization of silica-rich AIS sludge and polypropylene-based RMF under both normal and accelerated carbonation curing conditions. The AIS sludge, consisting predominantly of fine quartz particles, acts as an inert micro-filler that refines pore structure and enhances particle packing density, while its surface-bound moisture can slightly promote early-stage hydration. In parallel, the incorporation of RMF contributes to improved crack resistance and strain tolerance by bridging microcracks and mitigating brittleness. Furthermore, since the presence of polymeric fibers can enhance thermal stability and reduce conductivity, this study also explores the high-temperature resistance and residual strength of the composites after exposure to elevated temperatures. Through this integrated investigation, the present work provides new insights into the synergistic performance of mineral sludge and recycled polymer fibers, establishing a sustainable approach for producing durable, thermally stable, and carbon-mitigating cementitious composites.

## 2. Materials and Methods

### 2.1. Raw Materials Characterization

**Figure 1:**
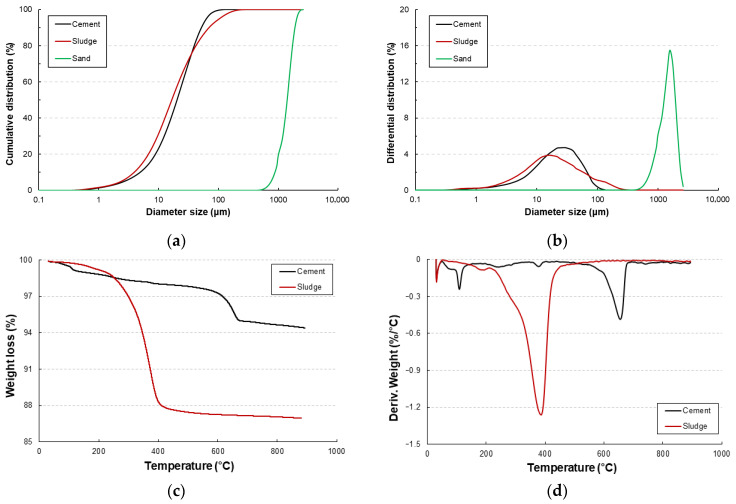
Particle size distribution and thermal characteristics of cement, sludge, and sand showing (**a**) cumulative distribution, (**b**) differential distribution, (**c**) TGA curves, and (**d**) DTG curves.

The AIS sludge used in this study was obtained as a by-product from a local quartz-based artificial stone manufacturer. The sludge contained approximately 27 wt.% moisture, determined following ASTM C 566 [20]. To minimize errors caused by moisture variation, the sludge was oven-dried at 100 ± 5 °C for 24 h to achieve a completely dry state before use. The dried sludge exhibited an off-white powdery appearance with particle sizes mainly between 10 and 15 µm. Laser particle-size analysis (Bettersizer 2600, Bettersizer Instruments, Dandong, Liaoning, China) showed D_10_ = 3.93 µm, D_50_ = 16.6 µm, and D_90_ = 172.7 µm, with a mean diameter of 29.0 µm. Compared with cement (D_50_ = 20.36 µm), the sludge displayed a slightly broader distribution, indicating a potential micro-filler effect (see Figure 1a,b). Thermogravimetric and differential thermogravimetric (TGA/DTG) analyses (TGA N-1000, Sinco, Seoul, Republic of Korea; SDT 650 Auto, TA Instruments, New Castle, DE, USA) indicated minor weight loss (≈12%) between 200 and 400 °C, attributed to the decomposition of residual organic polymers such as unsaturated polyester, polyvinyl alcohol, and polyacrylate used in the AIS fabrication process (see Figure 1c,d). Beyond 600 °C, the sludge remained thermally stable due to its high silica content [21,22].

**Figure 2:**
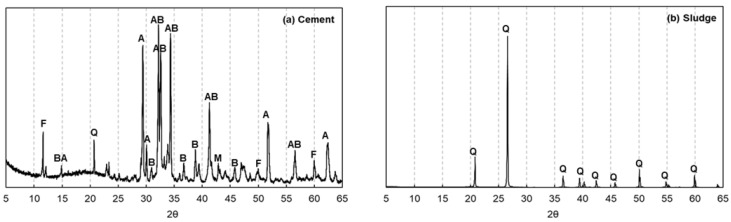
XRD patterns of cement and sludge: (**a**) Cement and (**b**) AIS sludge. In the cement pattern, A denotes alite (C_3_S), B indicates belite (C_2_S), AB represents overlapping peaks of aluminate/brownmillerite phases, F and BA correspond to ferrite (C_4_AF) and brownmillerite, respectively, and M denotes mayenite (C_12_A_7_). In the sludge pattern, Q indicates quartz (SiO_2_), which is the dominant crystalline phase.

In this study, X-ray diffraction (XRD) and X-ray fluorescence (XRF) analyses were performed to clarify the chemical composition of the AIS sludge and to assess its suitability as an additive in cement mortar. In the XRD patterns shown in Figure 2, the main peaks are denoted as follows: A represents alite, B represents belite, F represents brownmillerite, M represents monocarboaluminate, and Q represents quartz. As illustrated in Figure 2, the XRD results of the sludge exhibit broad and diffused peaks distributed mainly in the 10–40° (2θ) range, indicating the dominance of quartz as the principal crystalline phase. A diffraction pattern similar to that of hexagonal silica was also observed. In contrast, the polymeric components used during artificial stone fabrication are amorphous in nature and, therefore, do not produce distinct peaks in the XRD spectra.

**Figure 3:**
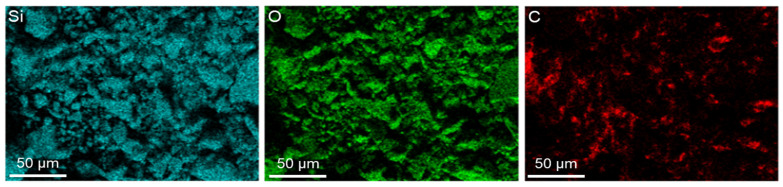
EDS elemental mapping of AIS sludge showing Si, O, and C distributions.

The energy-dispersive X-ray spectrometry (EDS) elemental mapping of the AIS sludge (Figure 3) shows a uniform distribution of Si and O, indicating the predominance of silica-based compounds within the particles. The particles exhibit mostly angular and irregular shapes, consistent with mechanically ground quartz fragments. A relatively low carbon signal was detected, suggesting that only a small amount of residual polymeric material remains from the artificial stone manufacturing process. These results confirm that the AIS sludge is primarily composed of SiO_2_-rich, inert mineral particles suitable for use as a micro-filler in cementitious composites.

According to the XRF results presented in Table 1, the AIS sludge is composed of more than 96.7% SiO_2_, exhibiting significantly lower contents of CaO and Al_2_O_3_ compared to cement. Considering this chemical composition, the sludge is unlikely to act as a reactive substitute for cement; instead, its high SiO_2_ content suggests a greater potential for use as a fine aggregate or inert filler within mortar mixtures.

**Figure 4:**
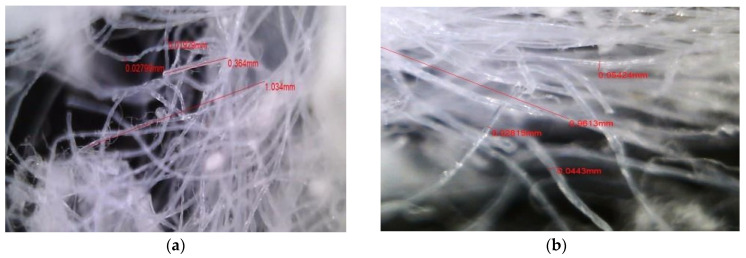
Magnified images of RMF showing (**a**) 250× and (**b**) 500× magnifications.

RMF were produced from commercially available polypropylene masks (Hydro KF80, Kidsmaru Co., Seongju, Republic of Korea). Prior to processing, ear loops and metal nose wires were removed. The mask sheets were then cut into small fragments measuring approximately 5–20 mm using scissors and a mechanical mixer (HR3760/00, Philips, Amsterdam, The Netherlands). The chopped mask pieces were mixed with 500 g of water and pulverized for 5 min to promote uniform separation of fibers. The resulting fibers were oven-dried at 60 °C for 24 h to remove residual moisture. The morphology and size distribution of the dried RMF were observed using an optical microscope, as illustrated in Figure 4, which shows irregularly shaped fibers with average diameters below 20 µm and lengths ranging from 0.5 to 2 mm.

### 2.2. Mix Design and Specimen Preparation

The mix proportions were determined based on previous studies on cementitious composites incorporating fine waste powders and polymer fibers [23,24]. In this study, ordinary Portland cement (Type I, Ssangyong C&E Co., Seoul, Republic of Korea) was used as the primary binder, and natural sand (Jumunjin Gyusa Co., Gangneung, Republic of Korea) served as the fine aggregate. The AIS sludge replaced 30 wt.% of the fine aggregate by dry weight, while RMF was added at 0, 0.25, 0.5, and 1.0 wt.% relative to the cement mass. The water-to-cement ratio (*w*/*c*) was fixed at 0.5 to maintain consistent workability among all mixtures. The detailed mix design is summarized in Table 2. The specimen notation is expressed as S for the sludge replacement ratio of fine aggregate and F for the fiber content relative to cement weight. For example, S30F0.5 refers to a mixture containing 30 wt.% AIS sludge and 0.5 wt.% RMF.

To ensure homogeneous distribution of both AIS sludge and RMF, the mixing process was performed in two stages. First, the cement and fine aggregate were dry-mixed for 3 min. Separately, water, sludge, and RMF were mixed for 3 min using a laboratory mixer to allow uniform dispersion of the fibers and sludge particles. The two mixtures were then combined and mixed for an additional 3 min to obtain a uniform mortar. The prepared mixture was cast into 50 × 50 × 50 mm molds for compressive strength testing and 40 × 40 × 160 mm molds for flexural testing. For each mixture, three identical specimens were prepared to ensure reproducibility and statistical reliability. All molds were covered with plastic film to prevent moisture loss and stored at 25 °C for 24 h.

After demolding, specimens were divided into two curing regimes: (1) Normal curing, performed at 25 °C and 60% relative humidity (RH); and (2) carbonation curing, conducted in a sealed chamber maintained at 25 °C, 60% RH, and 10% CO_2_ concentration (100,000 ppm). The curing durations were 7 and 28 days to evaluate both early and long-term properties. For each mix and curing condition, three specimens were tested, and average values were reported.

### 2.3. Experimental Procedures

A series of experimental tests was conducted to evaluate the physical, mechanical, and durability properties of mortar specimens incorporating AIS sludge and RMF under both normal and carbonation curing conditions (7 and 28 days). Density and thermal conductivity were measured to assess the compactness and thermal performance of the mortars. The bulk density was calculated from the oven-dried mass and specimen volume. Thermal conductivity was determined using a transient plane source analyzer (Hot Disk TPS 2500 S, Hot Disk Instruments, Göteborg, Sweden) at 25 °C and 60% RH. Three specimens were tested for each mix, and the mean values were reported.

Compressive and flexural strength were tested in accordance with ASTM C109 [25] and ASTM C348 [26]. After curing for 7 and 28 days, the specimens were loaded under displacement control using a UTM, and the maximum load at failure was recorded. Strength values were calculated from the recorded loads, and the averages of three specimens were reported. UPV (Ultrasonic Pulse Velocity) measurements were performed to evaluate the internal uniformity and compactness of the specimens after 28 days of curing. A portable UPV tester (Pundit PL-200, Proceq, Zurich, Switzerland; 54 kHz transducer) was used, and the average of three readings per specimen was taken. The carbonation area was examined using a 1% phenolphthalein indicator to visualize CO_2_ penetration. Split specimen surfaces were sprayed with the indicator solution, where non-carbonated zones appeared purple and carbonated zones remained colorless. The average carbonation area was obtained from three random measurements per specimen.

Fire-resistance tests were carried out to evaluate the residual mechanical performance of the mortars after exposure to elevated temperature. Specimens cured for 28 days were heated to 500 °C in an electric furnace (KDF 010 P, Denken-Highdental Co., Ltd., Kyoto, Japan) and maintained at that temperature for 2 h. After natural cooling to room temperature, compressive and flexural strengths were re-measured using the UTM. The reduction in strength was analyzed to assess the thermal stability of the carbonation-cured specimens compared with normally cured ones. TGA/DTG analyses were performed on powdered mortar samples to quantify the extent of carbonation and thermal decomposition. Samples were heated from room temperature to 1000 °C at 10 °C/min under a nitrogen atmosphere using a simultaneous thermal analyzer (SDT 650, TA Instruments, USA). Characteristic weight-loss peaks corresponding to dehydration, dehydroxylation, and decarbonation reactions were interpreted to confirm CaCO_3_ formation and overall carbonation efficiency [27].

## 3. Results and Discussion

**Figure 5:**
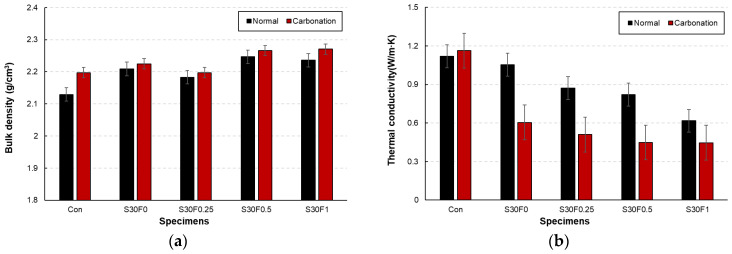
Bulk density and thermal conductivity of AIS sludge–RMF mortars under normal and carbonation curing conditions showing (**a**) bulk density and (**b**) thermal conductivity.

The bulk density and thermal conductivity of the AIS sludge–RMF mortars under normal and carbonation curing conditions are presented in Figure 5a,b, respectively. In general, all mixtures exhibited slightly higher densities after carbonation curing compared with normal curing. The increase in density is attributed to the formation of CaCO_3_ within the matrix during carbonation, which filled internal pores and refined the microstructure [28]. Among the specimens, S30F1 showed the highest density after carbonation curing, suggesting that the combination of 30 wt.% AIS sludge and 1.0 wt.% RMF contributed to pore refinement through both filler and fiber bridging effects. The thermal conductivity of the mortars exhibited an opposite trend to the density results. As shown in Figure 5b, the thermal conductivity decreased with increasing RMF content under both curing conditions, and the reduction was more pronounced after carbonation curing. This decrease is primarily due to the low intrinsic thermal conductivity of the polypropylene-based RMF and the additional pore formation around fiber interfaces. Moreover, the formation of CaCO_3_ during carbonation curing disrupted the continuous heat-transfer paths within the hardened matrix, further reducing thermal conductivity.

**Figure 6:**
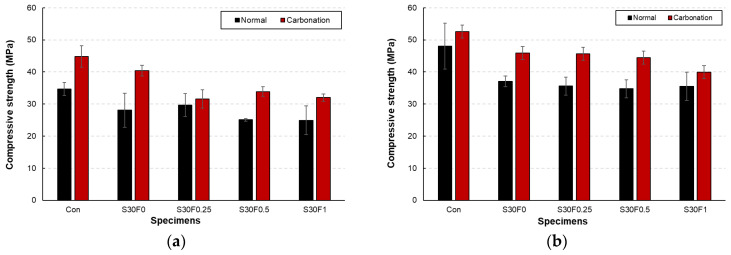
Compressive strength of AIS sludge–RMF mortars cured under normal and carbonation conditions showing (**a**) 7-day and (**b**) 28-day results.

The compressive strength results of AIS sludge–RMF mortars under normal and carbonation curing are presented in Figure 6a,b. For all mixtures, carbonation curing produced a noticeable strength enhancement compared with normal curing. After 7 days, the compressive strength of the carbonation-cured specimens increased by approximately 15–30%, while the 28-day strength exhibited an even greater improvement, reaching up to 28% higher than that of the normally cured specimens. This increase is mainly attributed to the formation of CaCO_3_ within the pore structure, which fills capillary voids and densifies the matrix. The Con specimen showed the highest overall strength in both curing regimes, but its relative gain from carbonation was smaller than that of the AIS- and RMF-modified specimens. In the mixtures containing AIS sludge, the fine SiO_2_-rich particles acted as micro-fillers, improving the particle packing and contributing to early-age strength recovery.

Meanwhile, the inclusion of RMF provided limited reinforcement because of its low stiffness and hydrophobic nature; however, when properly dispersed at moderate contents (0.25–0.5 wt.%), the fibers helped restrain microcrack propagation during carbonation, resulting in a synergistic effect with the CaCO_3_ formation. Excessive RMF content (1 wt.%) slightly reduced the compressive strength under both curing conditions, which can be ascribed to poor fiber dispersion and the creation of weak interfaces. Nevertheless, even at this dosage, the carbonation-cured specimen exhibited higher strength than its normally cured counterpart, confirming that carbonation curing effectively compensated for the loss of strength caused by increased porosity. These results demonstrate that combining 30 wt.% AIS sludge with an optimal RMF content of 0.25–0.5 wt.% yields a well-densified microstructure and significantly improved compressive performance.

**Figure 7:**
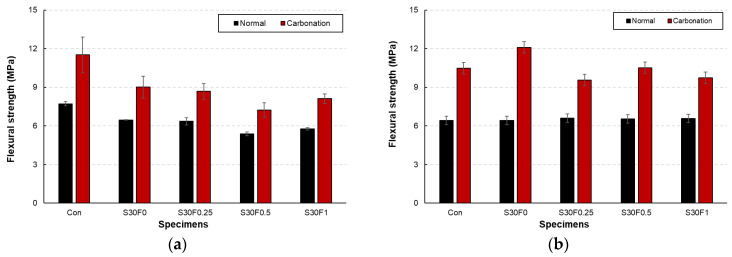
Flexural strength of AIS sludge–RMF mortars cured under normal and carbonation conditions showing (**a**) 7-day and (**b**) 28-day results.

The flexural strength of AIS sludge–RMF mortars under normal and carbonation curing is presented in Figure 7a,b. Similar to the compressive strength results, carbonation curing led to a significant increase in flexural strength across all mixtures. After carbonation, the flexural strength improved by approximately 60–90% compared with normal curing, indicating that carbonation enhanced the matrix stiffness and interfacial bonding between the cement paste and the solid particles. The generation of CaCO_3_ during carbonation is presumed to fill microvoids along the interfacial transition zone (ITZ), reducing microcrack propagation and improving overall flexural resistance. Under normal curing, the incorporation of RMF slightly decreased flexural strength with increasing fiber content. This reduction can be attributed to the hydrophobic nature of the polypropylene-based fibers, which leads to weak interfacial adhesion and potential fiber agglomeration. However, at moderate RMF contents (0.25–0.5 wt.%), the fibers acted as effective crack-bridging reinforcements, limiting crack widening and contributing to toughness improvement during carbonation curing. After 28 days of carbonation curing, all RMF-containing mixtures exhibited higher flexural strength than the corresponding normally cured specimens.

The S30F0.25 and S30F0.5 specimens, in particular, showed the most favorable balance between fiber dispersion and carbonation densification, achieving approximately 88% higher flexural strength than their normal-cured counterparts. Conversely, excessive fiber addition (1.0 wt.%) induced local defects and discontinuities in the matrix, resulting in marginal strength reduction. These results suggest that an optimal combination of 30 wt.% AIS sludge and 0.25–0.5 wt.% RMF effectively enhances the flexural performance through the synergistic effects of filler densification and fiber bridging. Although the compressive and flexural strength results were obtained from triplicate specimens with small standard errors, detailed statistical analyses such as ANOVA or t-tests were not performed in this study. This limitation will be addressed in future research to further improve the statistical robustness of the experimental results.

**Figure 8:**
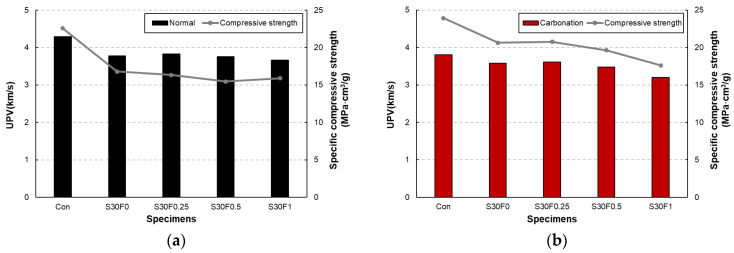
UPV and specific compressive strength of AIS sludge–RMF mortars at 28 days under (**a**) normal and (**b**) carbonation curing conditions.

The UPV and specific compressive strength of AIS sludge–RMF mortars at 28 days are presented in Figure 8a,b. In both curing regimes, UPV tended to decrease as the RMF content increased, indicating a slight reduction in internal compactness due to the inclusion of low-density polymeric fibers. The Con specimen exhibited the highest UPV, while the S30F1 specimen showed the lowest value under both curing conditions. This decline is attributed to the hydrophobic nature of RMF, which can hinder bonding at the ITZ and induce microvoids or weak interfaces that slow ultrasonic wave propagation. When comparing curing regimes, carbonation-cured specimens showed slightly lower UPV values than normally cured ones, despite their higher compressive strengths. This apparent discrepancy can be explained by the microstructural changes caused by carbonation. The formation of CaCO_3_ during carbonation curing promotes matrix densification; however, it can also lead to localized shrinkage and microcrack formation around reaction sites, which may interrupt the transmission of ultrasonic waves. The correlation between UPV and specific compressive strength suggests that while UPV is generally associated with matrix compactness, it may not directly represent mechanical performance in carbonation-cured systems. The carbonation reaction produces a complex balance between densification and internal stress, which enhances mechanical strength but slightly reduces acoustic continuity [29].

**Figure 9:**
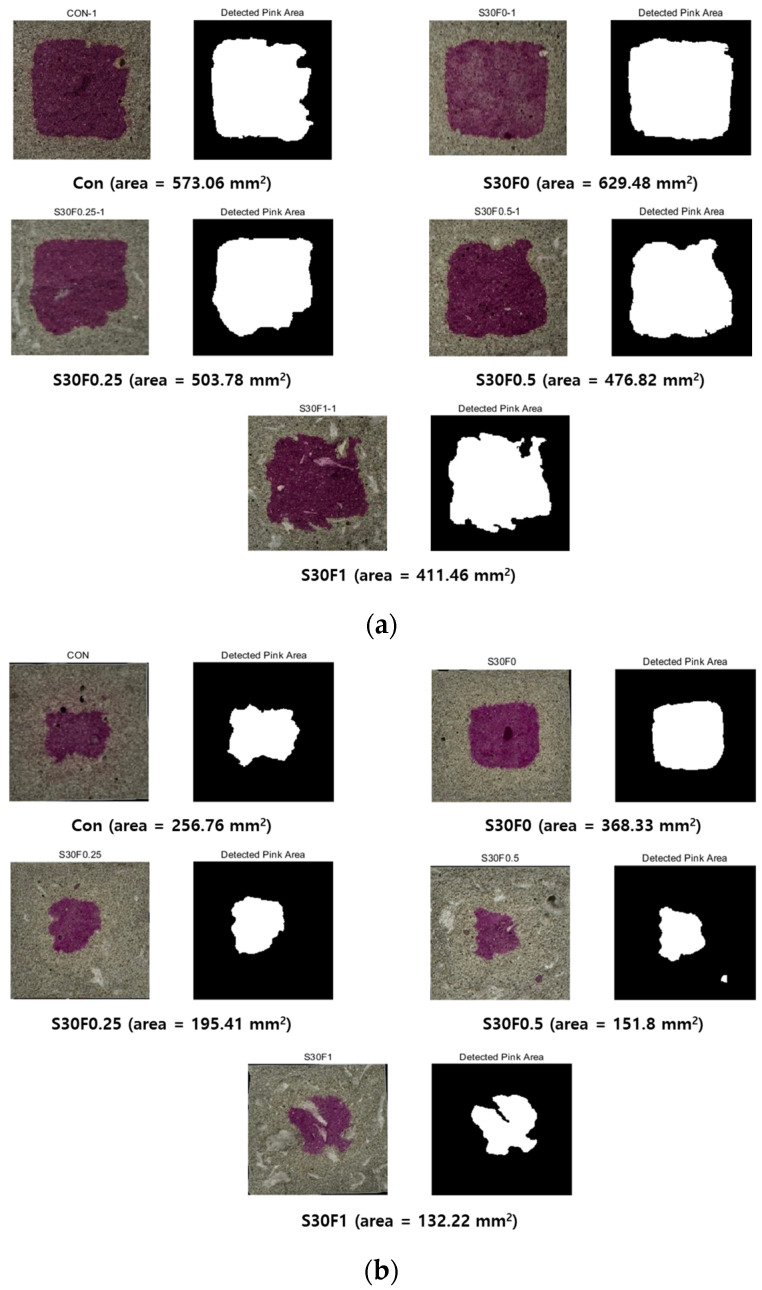
Carbonation area of AIS sludge–RMF mortars evaluated by phenolphthalein indicator after carbonation curing for (**a**) 7 and (**b**) 28 days.

The carbonation area of AIS sludge–RMF mortars was quantitatively analyzed using phenolphthalein indicator images, as shown in Figure 9a,b. The pink-stained region represents uncarbonated material, and the measured area indicates the remaining non-carbonated portion after 7 and 28 days of carbonation curing. A decrease in the pink area, therefore, corresponds to a greater degree of carbonation progress. After 7 days of carbonation curing (Figure 9a), the non-carbonated areas ranged from approximately 629 mm^2^ for S30F0 to 411 mm^2^ for S30F1. The results show that as RMF content increased, the uncarbonated area gradually decreased, suggesting enhanced CO_2_ diffusion due to the increased porosity and internal pathways introduced by the fibers. The presence of AIS sludge, composed primarily of fine SiO_2_ particles, contributed to maintaining matrix stability while allowing CO_2_ to penetrate evenly through the microstructure. After 28 days (Figure 9b), the overall carbonation areas were significantly reduced, with the remaining uncarbonated regions ranging between 368 mm^2^ (S30F0) and 132 mm^2^ (S30F1). The continuous reduction in pink area indicates the completion of carbonation in most specimens, particularly those containing higher RMF contents (≥0.5 wt.%). The formation of CaCO_3_ within pores and along the ITZ filled voids and refined the matrix structure, as also reflected in the increased compressive strength results (see Figure 6).

**Figure 10:**
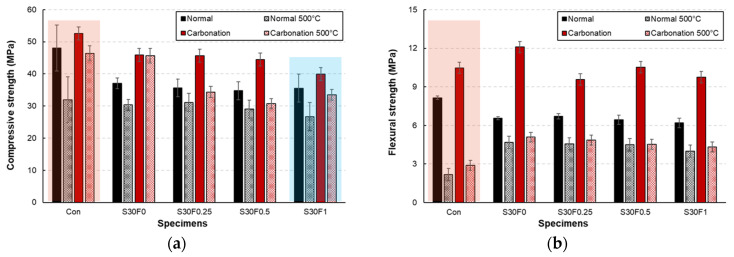
Residual compressive and flexural strengths of AIS sludge–RMF mortars after exposure to 500 °C showing (**a**) compressive strength and (**b**) flexural strength under normal and carbonation curing conditions. The red and blue shaded regions are included to emphasize the performance ranges of the control and RMF-modified mixtures.

The residual compressive and flexural strengths of AIS sludge–RMF mortars after exposure to 500 °C are presented in Figure 10a,b. Both strength parameters decreased after heating; however, carbonation-cured specimens consistently exhibited superior residual performance compared with those cured under normal conditions. This improvement can be attributed to the formation of CaCO_3_ within the matrix during carbonation curing, which densified the pore structure and acted as a thermal buffer against rapid heat transfer. For compressive strength (Figure 10a), the carbonation-cured mortars retained approximately 70–80% of their initial strength, while normally cured mortars maintained about 55–65%. The higher retention under carbonation curing resulted from the decomposition of CaCO_3_ into CaO and CO_2_ above 600 °C, which temporarily occupied pore spaces and delayed crack propagation. In contrast, normally cured mortars, lacking this secondary pore-filling mechanism, experienced severe dehydration of C–S–H and Ca(OH)_2_ decomposition near 450–500 °C, leading to interconnected microcracks and matrix softening. Among the mixtures, S30F0.25 and S30F0.5 showed the most stable performance, suggesting that moderate RMF addition effectively restrained thermal stress–induced cracking by distributing microstrain within the matrix.

The flexural strength results (Figure 10b) followed a similar trend but revealed distinct behavior due to fiber-matrix interaction. In the RMF-containing specimens, partial melting of polypropylene fibers occurred at around 160–170 °C, producing additional pore channels that alleviated internal vapor pressure during heating. This mechanism prevented explosive spalling and preserved matrix integrity despite localized strength reduction. After cooling, these microvoids acted as crack arrestors, contributing to improved post-heating toughness. The carbonation-cured S30F0.25 and S30F0.5 specimens maintained about 65–70% of their original flexural strength, whereas the normally cured specimens dropped below 50%. Excessive fiber incorporation (1 wt.%) led to excessive melting and coalescence of pores, resulting in decreased stiffness and a slight reduction in residual strength. The combined results demonstrate that the synergy between AIS sludge and RMF governs the thermo-mechanical stability of the mortar system. AIS sludge promotes a compact microstructure with enhanced heat conduction resistance, while RMF provides localized stress relaxation during thermal cycling. The carbonation-induced CaCO_3_ further reinforces this framework, enabling effective load redistribution after high-temperature exposure.

**Figure 11:**
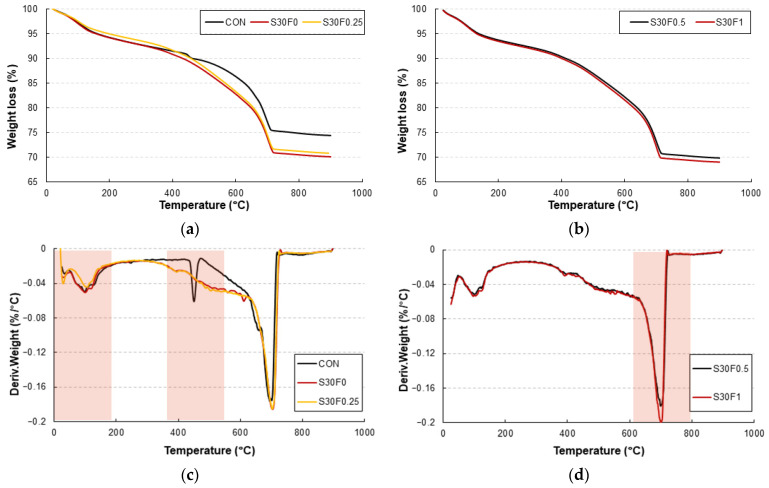
TGA and DTG results of AIS sludge–RMF mortars after carbonation curing for 28 days, showing (**a**) TGA curves for Con, S30F0, and S30F0.25, (**b**) TGA curves for S30F0.5 and S30F1, (**c**) DTG curves for Con, S30F0, and S30F0.25, and (**d**) DTG curves for S30F0.5 and S30F1. The red shaded regions highlight the major temperature intervals associated with characteristic decomposition reactions for visual reference.

The TGA and DTG results are shown in Figure 11a–d. Three major weight-loss regions were identified: (1) below 200 °C, corresponding to the evaporation of free and physically bound water; (2) between 200–450 °C, associated with dehydration of C–S–H gel and partial decomposition of polymeric RMF; and (3) from 600–800 °C, representing the decarbonation of CaCO_3_ formed during carbonation curing [30].

In Figure 11a,b the carbonation-cured mixtures exhibited a smaller overall mass loss than the control specimen, confirming their improved thermal stability. The incorporation of AIS sludge significantly moderated the mass-loss rate between 400 and 600 °C, indicating that the SiO_2_-rich particles acted as thermally inert fillers that restrained heat-induced decomposition. Similar findings were reported by Zhang et al. (2020) [5] and Park and Kim (2023) [24], who observed that silica-dominant fillers suppress C–S–H dehydration and mitigate microcrack growth under rapid heating. Consequently, the reduced weight loss of the AIS-containing mixtures explains the higher residual compressive and flexural strengths observed after 500 °C exposure.

The DTG curves in Figure 11c,d further clarify these behaviors. A distinct endothermic peak around 700 °C corresponds to the decomposition of CaCO_3_ to CaO and CO_2_, which is characteristic of carbonation-cured mortars. This peak was more pronounced in S30F0.25 and S30F0.5, verifying the higher carbonate content resulting from the accelerated carbonation reaction. During high-temperature exposure, partial CaCO_3_ decomposition temporarily releases CO_2_ within the matrix pores, which diffuses outward and compensates for internal vapor pressure. This mechanism delays explosive spalling and correlates with the relatively stable residual strength of carbonation-cured mortars shown in Figure 10. Minor DTG peaks between 250 and 400 °C are attributed to the thermal degradation of RMF. The polymeric fibers begin to soften and melt near 160–170 °C, generating fine channels that release vapor pressure and relax thermal stress. Although this process causes a small additional weight loss, it helps preserve matrix integrity at elevated temperatures [31]. The combination of these effects—CaCO_3_ decomposition buffering, silica-based thermal stability from AIS sludge, and stress relaxation by RMF—collectively explains the enhanced fire-resistance performance of the carbonation-cured specimens.

## 4. Conclusions

This study evaluated the mechanical, thermal, and carbonation characteristics of cement mortars incorporating AIS sludge and RMF under normal and carbonation curing conditions. The incorporation of AIS sludge effectively enhanced microstructural densification due to its SiO_2_-rich composition and fine particle size, which filled internal voids and improved matrix compactness. As a result, the AIS-containing mortars exhibited higher compressive strength and lower porosity, particularly under carbonation curing, where the formation of CaCO_3_ further refined the pore structure. Although this study focused on the mechanical, thermal, and carbonation characteristics of AIS sludge–RMF mortars, the influence of AIS sludge and RMF on workability was not evaluated. High fiber content, particularly at 1.0 wt.%, may reduce flowability and affect the uniform dispersion of fibers. Due to experimental constraints, flowability tests could not be performed; however, this is recognized as a limitation of the present work and will be addressed in future studies.

Carbonation curing considerably improved the overall mechanical performance compared with normal curing. The compressive and flexural strengths increased by up to 30%, attributed to the generation of stable CaCO_3_ and strengthening of the ITZ. The addition of RMF slightly reduced UPV but effectively lowered thermal conductivity, demonstrating its role in enhancing insulation and energy efficiency. When incorporated at moderate levels (0.25–0.5 wt.%), RMF provided effective crack bridging and stress distribution, leading to a balanced improvement in mechanical and thermal behavior.

Phenolphthalein tests confirmed that RMF promoted deeper carbonation by facilitating CO_2_ penetration, while AIS sludge maintained structural stability and limited excessive porosity. The combination of these two waste-derived materials resulted in uniform carbonation and efficient CO_2_ utilization. After exposure to 500 °C, carbonation-cured mortars retained up to 80% of their compressive and 70% of their flexural strength, indicating superior fire resistance. The TGA/DTG results supported these findings by revealing that CaCO_3_ formation, SiO_2_-rich AIS particles, and partially melted RMF collectively contributed to thermal buffering, vapor-pressure release, and structural cohesion at elevated temperatures. These results suggest that the combined use of AIS sludge and RMF can contribute to producing more sustainable and thermally stable cementitious materials by utilizing industrial waste and reducing CO_2_ emissions. Future work will focus on optimizing carbonation curing parameters and evaluating the long-term durability and field applicability of AIS–RMF-based mortars to verify their potential for practical use in low-carbon construction materials.

## Figures and Tables

**Table 1:** Chemical constituents of cement and AIS sludge determined by XRF (wt.%).

	MgO	Al_2_O_3_	SiO_2_	SO_3_	K_2_O	CaO	TiO_2_	Fe_2_O_3_
Cement	2.07	3.01	11.1	2.28	1.19	73.7	0.258	4.68
AIS Sludge	-	0.65	96.7	-	0.121	0.253	1.75	0.147

**Table 2:** Mix proportions of cement mortar incorporating AIS sludge and RMF.

Specimen	Cement	Fine Aggregate	AIS Sludge	Water	RMF
Con	100	200	0	50	0
S30F0		140	60		0
S30F0.25					0.25
S30F0.5					050
S30F1					1.00

## Data Availability

The original contributions presented in this study are included in the article. Further inquiries can be directed to the corresponding authors.

## Abbreviations

The following abbreviations are used in this manuscript:

AIS	Artificial interior stone
RMF	Recycled mask fibers
UPV	Ultrasonic pulse velocity
ITZ	Interfacial transition zone
